# Monoclonal Antibody RYSK173 Recognizes the Dinuclear Zn Center of Serum Carnosinase 1 (CN-1): Possible Consequences of Zn Binding for CN-1 Recognition by RYSK173

**DOI:** 10.1371/journal.pone.0146831

**Published:** 2016-01-22

**Authors:** Shiqi Zhang, Holger A. Lindner, Sarah Kabtni, Jaap van den Born, Stephan Bakker, Gerjan Navis, Bernard Krämer, Benito Yard, Sibylle Hauske

**Affiliations:** 1 Vth Department of Medicine (Nephrology/Endocrinology/Rheumatology), University Medical Center Mannheim, University of Heidelberg, Mannheim, Germany; 2 Department of Endocrinology, The first affiliated hospital of Anhui Medical University, Hefei, China; 3 Department of Anesthesiology and Surgical Intensive Care Medicine, University Medical Center Mannheim, University of Heidelberg, Mannheim, Germany; 4 Department of Nephrology, University Medical Center Groningen, University of Groningen, Groningen, the Netherlands; CNR, ITALY

## Abstract

**Background and Aims:**

The proportion of serum carnosinase (CN-1) recognized by RYSK173 monoclonal antibody negatively correlates with CN-1 activity. We thus hypothesized that the epitope recognized by RYSK173 is accessible only in a catalytically incompetent conformation of the zinc dependent enzyme and we mapped its position in the CN-1 structure. Since patients with kidney failure are often deficient in zinc and other trace elements we also assessed the RYSK173 CN-1 proportion in serum of these patients and studied the influence of hemodialysis hereon in relation to Zn^2+^ and Cu^2+^ concentration during hemodialysis.

**Methods and Results:**

Epitope mapping using myc-tagged CN-1 fragments and overlapping peptides revealed that the RYSK173 epitope directly contributes to the formation of the dinuclear Zn center in the catalytic domain of homodimeric CN-1. Binding of RYSK173 to CN-1 was however not influenced by addition of Zn^2+^ or Cu^2+^ to serum. In serum of healthy controls the proportion of CN-1 recognized by RYSK173 was significantly lower compared to end-stage renal disease (ESRD) patients (1.12 ± 0.17 vs. 1.56 ± 0.40% of total CN-1; p<0.001). During hemodialysis the relative proportion of RYSK173 CN-1 decreased in parallel with increased serum Zn^2+^ and Cu^2+^ concentrations after dialysis.

**Conclusions:**

Our study clearly indicates that RYSK173 recognizes a sequence within the transition metal binding site of CN-1, thus supporting our hypothesis that metal binding to CN-1 masks the epitope. The CN-1 RYSK173 proportion appears overall increased in ESRD patients, yet it decreases during hemodialysis possibly as a consequence of a relative increase in transition metal bound enzyme.

## Introduction

Serum carnosinase (CN-1) (UniProt identifier Q96KN2) is abundantly expressed in the liver from where it is secreted into the circulation [1]. Based on structural similarity, CN-1 has been classified as metallopeptidase belonging to the M20 family of clan MH. CN-1 is composed of two structural domains of which one adopts an α/ß/α sandwich fold that features a dinuclear zinc-binding site [2]. The other, smaller domain is inserted into the middle of the metal-binding domain and, as in most M20 family enzymes, mediates homodimerization of CN-1. Two active sites per dimer are located at the interface between one metal-binding domain and the two associated dimerization domains, respectively. In CN-1 (MEROPS accession number MER015142), H478 and E200 chelate zinc 1, and H132 and D228 chelate zinc 2. D165 acts as a bridging ligand and the catalytic water molecule completes the tetrahedral coordination sphere for both zinc ions. Mutation of H132, D165, or E200 would lead to the loss of CN-1 activity, indicating the importance of metal-binding for enzyme activity [3]. Previously we have demonstrated that serum CN-1 concentration and activity are genetically determined by the (CTG)_n_ polymorphism [4, 5] and by N-glycosylation of CN-1 [6]. In addition CN-1 hydrolytic activity can be modulated by divalent metal ions, such as Cd^2+^, Co^2+^, Fe^2+^, Ni^2+^ [3], and by competing substrates, such as anserine and homocarnosine [1, 7].

In the past years the *CNDP1* gene, encoding CN-1, has attracted much attention as susceptibility locus for diabetic nephropathy (DN) in type 2 diabetic patients [4, 8]. It is believed that genotypes that are associated with low serum CN-1 concentrations may afford protection against DN as a consequence of reduced carnosine degradation. Yet, it should be emphasized that irrespective of the *CNDP1* genotype carnosine concentrations are extremely low or undetectable in human serum or plasma. Carnosine can be detected in serum only transiently after oral carnosine supplementation in individuals with low serum CN-1 concentrations [9].

We have developed two ELISA assays for detection of human serum CN-1 [10]. Quantitative assessment of serum CN-1 concentrations using the ATLAS monoclonal antibody based ELISA, reveals a good correlation with CN-1 activity. The other ELISA is based on the so-called RYSK173 monoclonal antibody and only detects a certain proportion of the total serum CN-1 concentration. The RYSK173 proportion can be increased by addition of EDTA or serum denaturation [10]. Hence the RYSK173 based ELISA assesses CN-1 quality rather than quantity. While in the majority of individuals the proportion of total CN-1 that is recognized by RYSK173 is low (0.1 to 2%), we have reported that individuals with a high proportion of this conformation (>15%) have low CN-1 activities [10]. Since metal ions at the active center of CN-1 are contributing to its enzyme activity, the proportion of CN-1 that is recognized by RYSK173 might be partly lacking these ions. Because formal proof for the assumption that RYSK173 distinguishes between apoenzyme and transition metal bound CN-1 is lacking, we sought to probe the position of the RYSK173 epitope in relation to the metal binding site of CN-1.

## Methods and Materials

### Generation of RYSK173 antibody

Monoclonal antibody RYSK173 was generated as described in [10]. In brief, Balb/c mice were immunized by intraperitoneal injection of recombinant human CN-1. Three days after the last boost splenocytes were collected and fused with SP2/0 myeloma cells. The fused cells were seeded in 96-well plates (1×10^3^ cells per well) and screened for anti-carnosinase antibody in the supernatant by indirect immune fluorescence (IIF) on CN-1 transfected COS7 cells. Positive wells were sub-cloned by limiting dilution. Clone RYSK173 was selected on the basis of a strong staining in IIF.

### Cell culture and transfection

COS7 cells (Invitrogen, Karlsruhe, Germany) were cultured in Dulbecco´s modified Eagle´s medium (DMEM/F-12, GlutaMAX(TM), Invitrogen, Karlsruhe, Germany) supplemented with 10% fetal calf serum (FCS) and 1% penicillin/streptomycin at 37°C and 5% CO_2_. The cells were transfected with various length of *CNDP1* constructs and lipofectamine2000 (Invitrogen, Karlsruhe, Germany) according to the manufacture´s instruction. Five hours after transfection, the medium was replaced by normal DMEM medium. Supernatants and cell lysates were collected after 48 hours. Cells were lysed on ice by lysis buffer with the addition of dithiothreitol (Fluka Chemie GmbH, Buchs, Germany), protease inhibitor (Roche, Mannheim, Germany), and phosphatase inhibitor (Sigma, Steinheim, Germany). Cell lysates were centrifuged for 10 minutes (14000rpm, at 4°C) to remove cell debris.

Human umbilical vein endothelial cells (HUVEC) were isolated from fresh human umbilical cords as previously described [11]. The cells were cultured in endothelial cell growth medium enriched with 2% FCS and 50ng/ml amphotericin B together with 50μg/ml gentamicin at 37°C and 5% CO_2_. The flasks for HUVECs were coated in 1% gelatin 20 minutes before use. All experiments involving HUVECs were performed at passages 2–6. Umbilical cords were obtained from donors in the obstetric department of Medical Center Mannheim. This was also approved by local ethics committee (Medizinische Ethikkommission II der Medizinischen Fakultät Mannheim) and all donors gave their written informed consent (No. 2015-518N-MA).

### Lentivirus production and transduction

Lentiviral transduction was performed to produce HUVECs with stable CN-1 expression. Human *CNDP1* cDNA (RZPD, Library 983, entry No.BX094414) was firstly constructed into lentivirus based vector ppM337 which was kindly provided by Prof. P. Maier from Radiology department of Medical Faculty Mannheim of Heidelberg University. In brief, each culture dish was coated with 0.1mg/ml poly-D-lysine (Sigma, Steinheim, Germany) for 5 min and then washed with sterile distilled water. 5×10^6^ HEK293T/17 cells were seeded per dish in DMEM. For each dish, 4.4μg lentiviral plasmid, 3.4μg packaging plasmid pCMV891 and 2.2μg pMD.G were added to cells with 46μl metafectene (Biontex, Munich, Germany). Cell medium was substituted by 14ml DMEM with 10mM Na-butyrate for 8 hours the next day. Lentiviruses containing cell medium was collected and concentrated using Vivaspin20 (Sartorius stedim, Göttingen, Germany) on the third day.

For transduction, HUVECs were incubated in 1:100 diluted virus containing HEK293T/17 supernatant for 48 hours. Cells were lysed either by lysis buffer or by a freeze and thaw cycle in liquid nitrogen. Westernblotting of 20μg total protein from HUVECs supernatant and cell lysates was performed to confirm the transduction.

### Construction of *CNDP1* variants

*CNDP1* cDNA was taken as a template to generate *CNDP1* fragments by PCR. PCR amplification was carried out from bp 1 to bp 312, from bp 1 to bp 471 and from bp 313 to bp 471 using the following forward and reverse primers respectively:

1–312: cagcccatcgatatggatcccaaactcaggaga and agctttaaatcgatgatcgggcagctgctgag

1–471: cagcccatcgatatggatcccaaactcaggaga and agctttaaatcgatgtttcccgtctacctccgtcagc

313–471:cagggatccatgggtcagagtcttccaatacctcccg and agctttaaatcgatgtttcccgtctacctccgtcagc.

Amplification products were cloned into the pCSII + mt vector providing a N-terminal 6×myc-tag and subsequently transfected into COS7 cells as previously described.

### Synthetic peptides

Three overlapping CN-1 peptides were synthesized (BIOMATIK, Wilmington, USA) that completely covered the coding sequence from bp 313 to bp 471:

313–391: CN1-1 (DGQSLPIPPVILAELGSDPTKGTVCF),

347–440: CN1-2 (LAELGSDPTKGTVCFYGHLDVQPADRGDGWL),

393–471: CN1-3 (HLDVQPADRGDGWLTDPYVLTEVDGK).

Fine epitope mapping was subsequently performed using two peptides that covered the overlapping sequence of CN1-2 and CN1-3, i.e. CN1-4 (HLDVQPAD) and CN1-5 (PADRGDGWL). All peptides were dissolved in sterile distilled water (final concentration: 10mg/ml) and were tested for recognition by RYSK173 by dotblot analysis and/or ELISA.

### CN-1 detection

Both the RYSK173 and ATLAS based CN-1 ELISA assays were used for detection of CN-1 in serum samples. CN-1 ELISAs were performed as described [10]. For epitope mapping synthetic peptides were directly coated on the ELISA plates.

Gel electrophoresis and western blot were performed using a standard protocol. Dot blot assays were performed by spotting 2 μl of synthetic peptide solution on a nitrocellulose membrane. The membrane was dried and processed similar as has been described for westernblotting [10].

### Site mutagenesis

One nucleotide mutation in CN-1 was generated by the QuikChange^™^XL site-directed mutagenesis kit (Stratagene, Waldbronn, Germany). H132 was exchanged to Q132. The forward and reverse primers were as follows. The sequence of the mutant *CNDP1* was confirmed by DNA sequencing.

Forward primer: gcttctacggccagttggacgtgcagc

Reverse primer: gctgcacgtccaactggccgtagaagc

### Patients

Thirty one patients on hemodialysis were recruited from our dialysis ward. They all had different causes of end stage renal disease. Renal transplant recipients, patients with urinary tract infection or fever at the time of investigation were excluded. Diabetic patients were also excluded since diabetes *per se* might potentially influence CN-1 metabolism. Demographic and relevant clinical data of these patients are shown in Table 1. Sera from age and gender matched healthy controls (n = 111) were retrieved from our bio-bank. The study was approved by local ethics committee (Medizinische Ethikkommission II der Medizinischen Fakultät Mannheim) and all patients gave their written informed consent prior to study (No. 0193/2001).

**Table 1:** Relevant clinical data of ESRD patients.

ESRD patients (n = 31)	
Gender (male)	20 (65%)
Age (year)	55.0 ± 2.4
Plasma creatinine (mg / dl)	10.6 ± 0.58
[Zinc] before/after hemodialysis (μmol/L)	9.5 ± 0.2 / 10.9 ± 0.2 (p < 0.05)
[Copper] before/after hemodialysis (μg/L)	14.1 ± 0.4 / 15.3 ± 0.4 (p < 0.05)
Duration of dialysis (months)	81 ± 82
Causes of disease	
Glomerulonephritis	15
Vascular disease	4
Pyelonephritis or hydronephrosis	6
Polycysitic kidney disease	2
Others (non-diabetes)	4

### Trace metal analysis

Copper and zinc in serum were analysed at Laboratory Limbach (Heidelberg, Germany) by colorimetric photometry as described previously [12, 13]. Visibly hemolyzed serum samples were not analysed.

### Statistical analysis

Quantitative data are depicted as mean ± SD. Student t test (normal distribution) or Wilcoxon-Mann-Whitney test (non-normal distribution) was used to compare differences between the groups. Correlation between values was evaluated by Pearson correlation coefficients. Significance was defined according to a p-value < 0.05. Statistical analysis was performed with GraphPad Prism 6.0 (GraphPad Software, Inc, La Jolla, California).

## Results

### Epitope mapping of RYSK173

**Fig 1:**
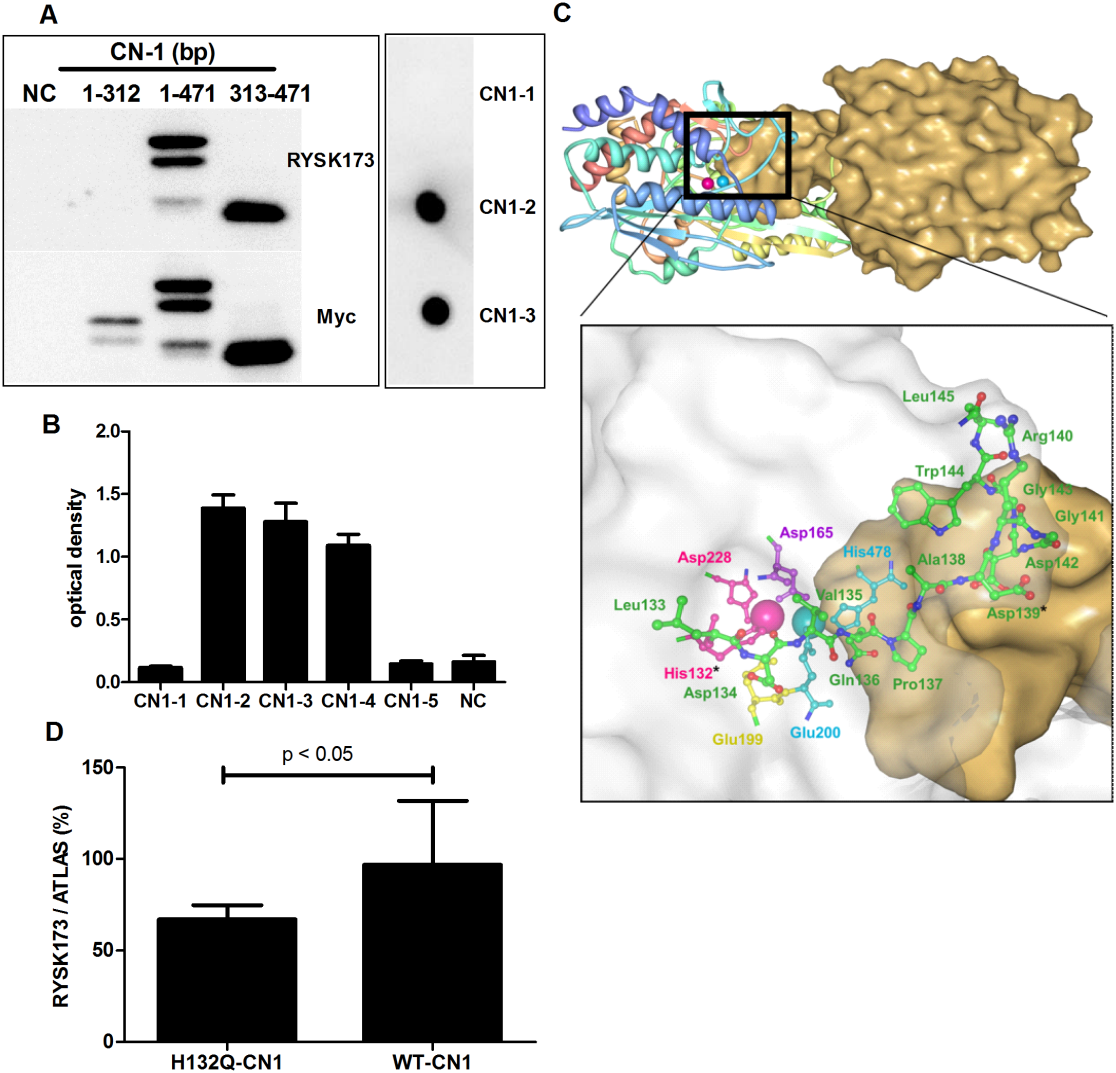
Epitope mapping of RYSK173. A: Myc-tagged recombinant CN-1 fragments were expressed in COS7 cells by transfection. Cells were harvested and the recombinant proteins were detected by Westernblotting using RYSK173 or anti-Myc (Fig on the left). Three overlapping synthetic peptides (CN1-1, CN1-2 and CN1-3) corresponding to bp 313–471 were tested in dotblot analysis (Fig on the right). B: Two additional peptides (CN1-4 and CN1-5) corresponding to the overlapping sequence of CN1-2 and CN1-3 were tested in ELISA for recognition by RYSK173. C: Location of the dinuclear zinc center at the CN-1 homodimer interface. The monomer on the left is depicted as a ribbon drawing and the monomer on the right is rendered as solid surface. Zinc1 and zinc2 in the left monomer are shown as cyan and magenta colored spheres, respectively. The enlarged view details the position of the RYSK173 epitope, which is confined to the amino acid sequence H132 to D139 (both marked with an asterisk). The surface of the left subunit is rendered semi-transparent. The Fig was made with Protein Workshop. D: H132 was exchanged for glutamine (Q) by site directed mutagenesis. The recombinant proteins were detected in transfected cell lysates using the ATLAS and RYSK173 based ELISA. The results are expressed as RYSK173/ATLAS ratio x 100%. NC: negative control, WT: wild type.

Since we have previously demonstrated that addition of EDTA improved CN-1 detection by RYSK173 [10], we anticipated that the epitope on CN-1 which is recognized by RYSK173 resides within the metal-binding domain of CN-1. Because we also have shown that RYSK173 recognizes a CN-1 fragment when truncated before the first N-glycosylation site [6] we constructed three myc tagged CN-1 fragments that differed in size (Fig 1). While a myc-tagged recombinant fragment of CN-1 spanning bp 1–471 was detected by both RYSK173 and myc antibodies, a shorter fragment (bp 1–312) was only recognized by the myc antibody. Because a recombinant CN-1 fragment corresponding to bp 313–471 was also recognized by both antibodies our data suggest that the RYSK173 epitope resides within this part of the CN-1 protein (Fig 1A, panel on the left). Three overlapping synthetic peptides of approximately 30 amino acids that covered the 313–471 bp fragments were subsequently tested by dot blot analysis for RYSK173 binding. CN1-2 and CN1-3 were clearly recognized whereas CN1-1 was not (Fig 1A, panel on the right). To delineate the exact epitope on CN-1 that is recognized by RYSK173, two different peptides corresponding to the overlapping sequence of CN1-2 and CN1-3 (HLDVQPADRGDGWL) were tested by ELISA. The peptides CN1-4 (HLDVQPAD) and CN1-5 (PADRGDGWL) consisted of either the first 8 or last 9 amino acids of the overlapping peptide sequence of CN1-2 and CN1-3 respectively. Synthetic peptides CN1-2, CN1-3 and CN1-4 were all detected in ELISA by RYSK173 whereas CN1-1 and CN1-5 were not (Fig 1B). This demonstrates that the RYSK173 epitope is contained in the sequence spanning the zinc 2-binding residue H132 to D139. Inspection of its spatial position within the metal binding domain of CN-1 reveals its proximity to both metal ions and the homodimer interface (Fig 1C). We also demonstrate that H132 is an integral constituent amino acid of the RYSK173 epitope, since site directed mutation of H132 to Q132 in recombinant CN-1 (rCN-1) expressed in COS7 cells resulted in a significant decrease in detection by RYSK173 (Fig 1D).

### Recognition of serum CN-1 and recombinant expressed CN-1 in HUVECs by RYSK173

**Fig 2:**
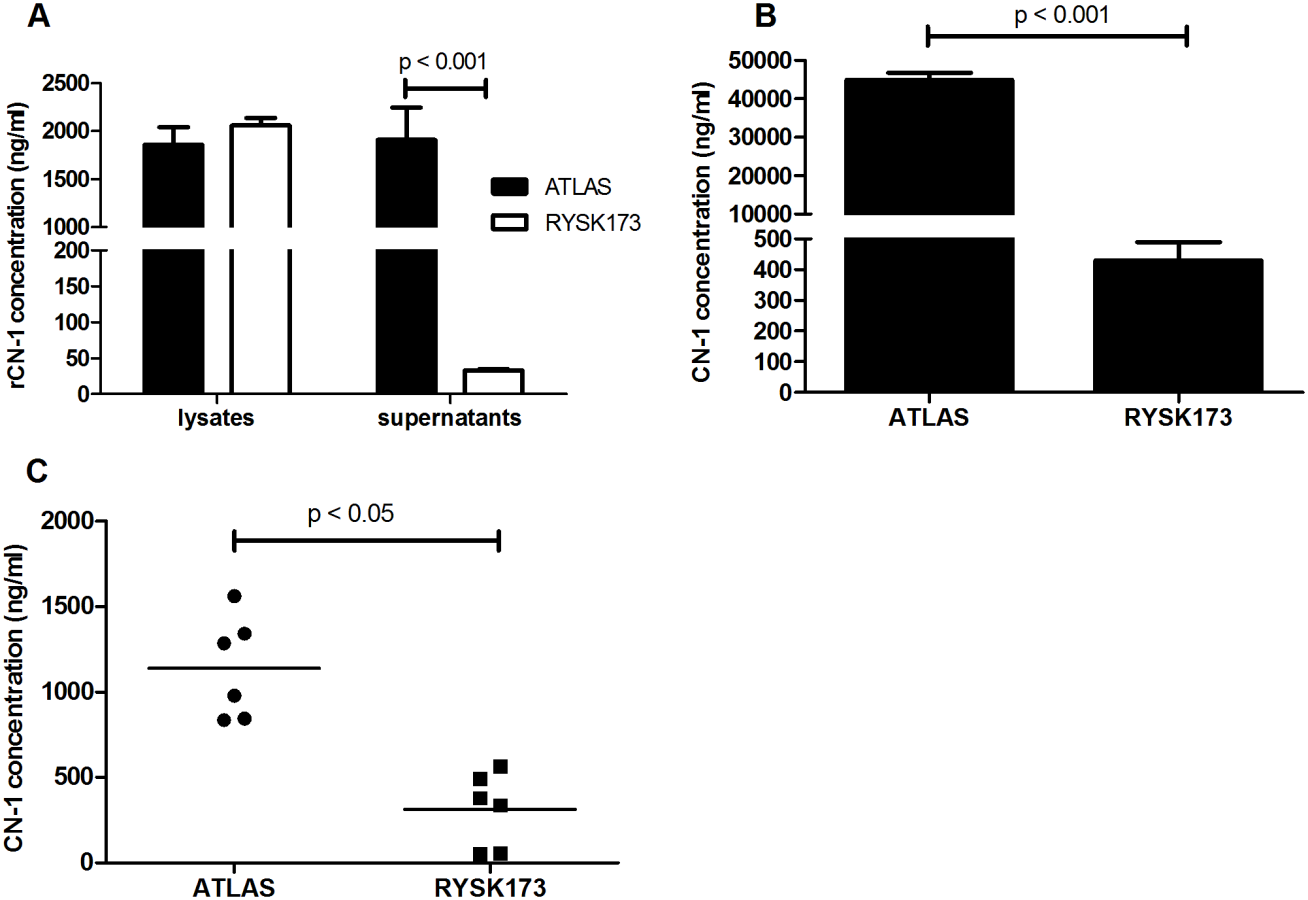
Recognition of recombinant and serum CN-1 by RYSK173. A: recombinant CN-1 was expressed in HUVECs by lentiviral transduction. The recombinant proteins were detected in cell lysates and supernatants using the ATLAS (filled bars) and RYSK173 (open bars) based ELISA. The results of 6 transduction experiments are depicted and expressed as mean CN-1 concentration (ng/ml) ± SD. B: Serum samples of 111 healthy individuals were tested in the ATLAS and RYSK173 based ELISA. C: Cell disruption was performed by repeated freeze thawing. Similar as in serum RYSK173 detected significantly lower amounts of CN-1.

Since our epitope mapping studies have demonstrated that epitope which is recognized by RYSK173 directly contributes to the formation of the Zn dinuclear centres in CN-1, we studied if CN-1 recognition by RYSK173 is changed once CN-1 is secreted. This would indicate that loading of the Zn centres would occur extracellularly. To this end, we first assessed if ectopically expressed rCN-1 in supernatants and cell lysates of CN-1 transduced HUVECs is recognized in the RYSK173- and ATLAS based ELISA, the latter recognizing total CN-1 [10]. While in cell lysates of *CNDP1* transfected HUVECs (n = 6) CN-1 was detected to a similar extent by ATLAS and RYSK173, in supernatants only a fraction of total CN-1 was recognized by RYSK173 (Fig 2A). Since the lysis buffer contained both EDTA and DTT, the difference in RYSK173 recognition of CN-1 in cell lysates and supernatants is likely explained by denaturation of the protein [10]. We therefore used freeze thaw cycles as a more gentle way to obtain intra-cellular proteins (n = 6). Although the yield of CN-1 was significantly lower than the previous cell lyses method, similar as observed for serum (Fig 2B), CN-1 concentrations were significantly higher in the ATLAS based as compared to the RYSK173 based CN-1 ELISA (Fig 2C), albeit that the difference was less pronounced as compared to serum.

**Fig 3:**
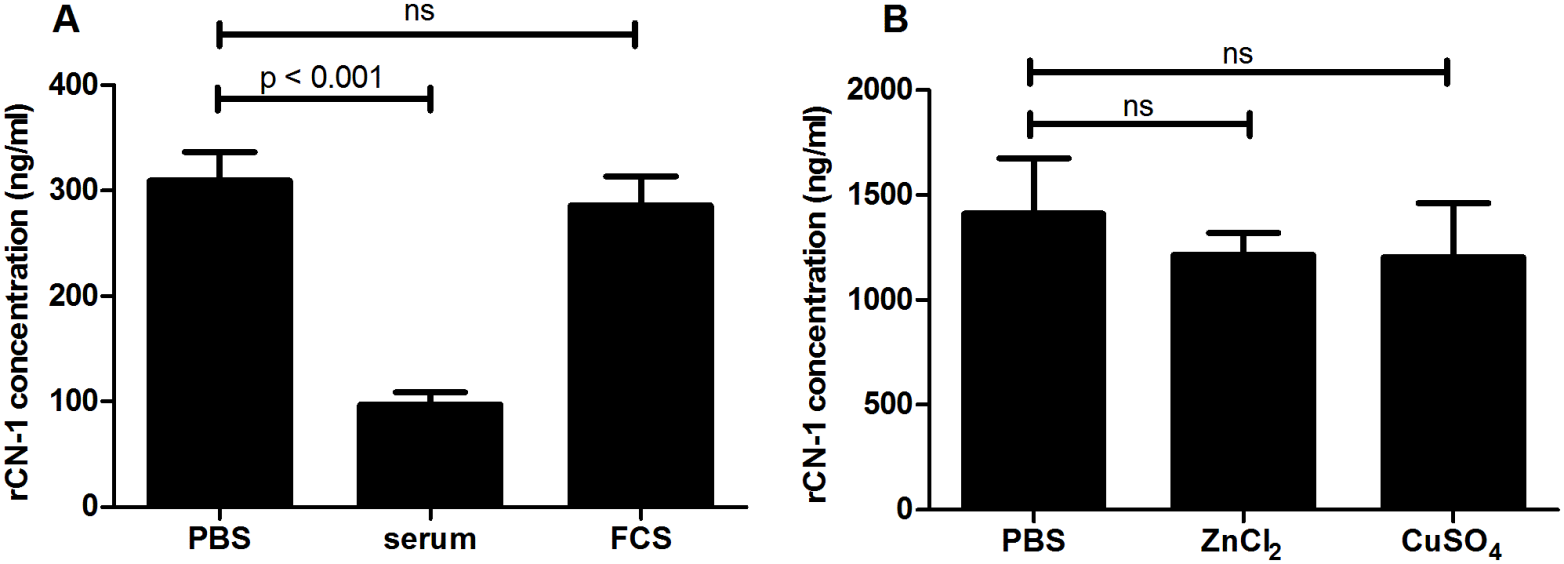
Recovery of recombinant CN-1 in serum. A: Equal amounts of recombinant CN-1 were spiked in PBS, human serum or FCS. While in human serum detection of CN-1 by RYSK173 was strongly diminished this was not observed in FCS. B: The influence of ZnCl_2_ (100μM) and CuSO_4_ (100μM) on detection of CN-1 by RYSK173 was tested. Ns: not significant.

We next tested if recovery of rCN-1 was impaired when spiked in human serum or FCS. While the recovery of rCN-1 significantly decreased in human serum, detection of rCN-1 was not significantly influenced in FCS (Fig 3A). Because the concentrations of the trace metal ions Zn^2+^ and Cu^2+^ were different between the two types of serum (Zn^2+^ concentration: 11.7±0.1 vs. 18.7±0.1 μmol/L and Cu^2+^ concentration: 822±11.3 vs. 89.5±0.7 μg/L; human serum vs. FCS), we tested if addition of ZnCl_2_ or CuSO_4_ influences the recovery of rCN-1 in the RYSK173 based ELISA. Neither addition of 100μM ZnCl_2_ nor CuSO_4_ were able to reduce the recovery of rCN-1 in PBS (Fig 3B) or in FCS (data not shown). Additionally, no significant differences were found even if higher concentrations of metal ions were used (data not shown).

### The relative proportion of CN-1 recognized by RYSK173 is increased in ESRD patients

**Fig 4:**
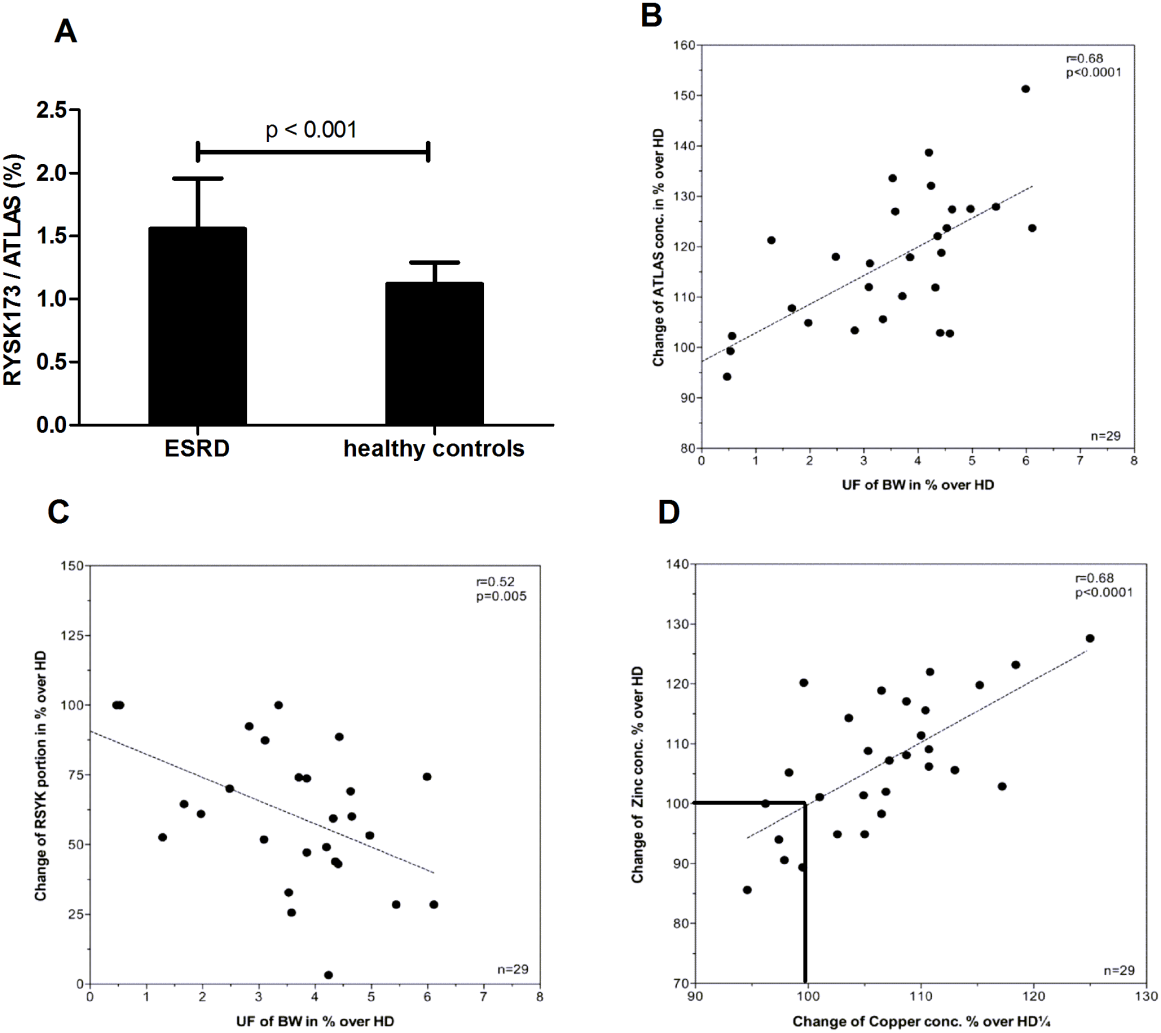
Serum CN-1 concentrations in ESRD patients. A: Sera of 31 ESRD patients were collected and tested in the ATLAS based and RYSK173 based ELISA. Sera of 111 healthy individuals served as control. The results are expressed as RYSK173/ATLAS ratio x 100%. B: Serum was collected directly before or after hemodialysis. The influence of hemodialysis on total CN-1 expression (B), the RYSK173 proportion (C) and changes in Zn^2+^ and Cu^2+^ (D) concentrations were assessed. UF: ultrafiltration, BW: body weight, HD: hemodialysis, conc: concentration.

Recent evidence indicates that patients with kidney failure are often deficient in zinc and other trace elements [14]. We therefore assessed to what extent end-stage renal disease (ESRD) patients differ in the proportion of CN-1 that is recognized by RYSK173. As depicted in Fig 4A, the relative proportion of CN-1 that was recognized by RYSK173 was significantly higher in ESRD patients as compared to healthy controls. The serum Zn^2+^ concentrations in ESRD patients ranged from (6.8 to 12.1 μmol/L) while that of healthy controls were all in the normal range (9.2 to 18.4 μmol/L). Since all ESRD patients were on hemodialysis, we also assessed the influence of hemodialysis on serum CN-1 concentrations in parallel to changes in serum Zn^2+^ and Cu^2+^ concentrations. To this end, serum CN-1, measured by both RYSK173 and ATLAS based ELISA, Zn^2+^ and Cu^2+^ concentrations were assessed directly before and after one hemodialysis session. While total serum CN-1 concentrations were significantly increased in the post-dialysis samples and correlated to the amount of ultrafiltration (Fig 4B), the proportion of CN-1 recognized by RYSK173 was decreased in these samples (Fig 4C). Zinc and copper concentrations were expressed as percentage (%) change of Zn^2+^ and Cu^2+^ concentrations in the post dialysis sample relative to the serum sample obtained directly before hemodialysis. In approximately 5 out of 31 samples the Zn^2+^ and Cu^2+^ ion concentration did not change or decreased over hemodialysis (Fig 4D, lower left quadrant), while in the majority of samples either Zn^2+^, Cu^2+^ or both were increased after dialysis (Fig 4D).

## Discussion

In the present study we sought to map the epitope that our anti-CN-1 monoclonal antibody RYSK173 recognizes in CN-1. Our previous finding that chelators of transition metal ions were able to unmask the epitope had led us to expect its location in the vicinity of the metal binding site of CN-1. Our results unambiguously demonstrate that the epitope recognized by RYSK173 indeed is in intimate contact with the dinuclear zinc site in CN-1 and even includes metal ligand H132. Metalloproteases are ubiquitous enzymes able to degrade an array of protein substrates. Mostly these enzymes utilize conserved amino acid residues to generate a scaffold capable of binding one or two metal ions [15]. Their functionality depends on subtle interactions between the electronic properties of the metal ion, dictated by its coordination chemistry, and the stability of protein conformations.

Previous mutational and structural analyses of CN-1 homologs from the M20 family have suggested that these enzymes require both metal sites in their dinuclear zinc centers for optimal protein stability but that zinc 1 is often bound with low affinity [16]. It may thus be easily removed by EDTA, a treatment which we have formerly shown to partially improve CN-1 recognition by RYSK173 [10]. Immediate proximity of the epitope to both zinc sites in CN-1 (Fig 1) agrees with the notion that removal of zinc 1 leads to its exposure due to local protein unfolding. The dinuclear zinc center in CN-1 is formed by H478 and E200 which coordinate with zinc 1 and H132 and D228 coordinating with zinc 2. Site directed mutagenesis of H132 to glutamine significantly impaired binding of RYSK173 indicating that this residue is part of the RYSK173 epitope. It should be emphasized that binding of RYSK173 in the presence of EDTA is still significantly lower compared to binding of the polyclonal antibody ATLAS. Only after protein denaturation binding of RYSK173 to CN-1 equals that of ATLAS. This is in line with our experimental data that no difference in CN-1 binding was observed between RYSK173 and ATLAS in lysates of *CNDP1* transduced HUVECs when a DTT and EDTA containing lysis buffer was used.

In line with our previously published data [10], a large difference in CN-1 concentrations are detected in human serum when using the ATLAS or RYSK173 based ELISA (Fig 2B) respectively. This might be explained by the fact that in a fraction of CN-1 the Zn centers are not completely occupied. To elucidate if Zn binding already occurs inside the cells shortly before CN-1 is secreted, we performed ELISA on supernatants and cell lysates using the RYSK173 antibody. Indeed we found a significant lower amount of CN-1 in the RYSK173- as compared to the ATLAS based ELISA in cell lysates (p < 0.05, Fig 2C) that were obtained without the use of denaturation or EDTA. This might indicate that zinc binding to CN-1 already occurs intracellularly and still proceeds extracellularly as the difference between the RYSK173 and ATLAS based ELISA was much more pronounced in supernatants (p < 0.001, Fig 2A) as compared to cell lysates. The ATLAS and RYSK173 based ELISA detected comparable CN-1 concentrations in cell lysates when the lysis buffer contained DTT and EDTA.

The finding that recovery of rCN-1 by the RYSK173 based ELISA was impaired in human but not in fetal calf serum remains unexplained in our study. While Zn^2+^ concentrations were slightly different in these two types of serum, the Cu^2+^ concentration in FCS was only 10% of that found in human serum. Although it has been suggested for bovine lens leucyl aminopeptidases (blLAP) [17] and the aminoacylhistidine dipeptidase (*PepD*) [18, 19], that the zinc centers might be exchanged by other divalent cations with different exchange kinetics, to our knowledge this has not been reported for CN-1. Addition of CuSO_4_ did not impair detection of rCN-1 in the RYSK173 based ELISA, suggesting that binding of Cu^2+^ to CN-1 in human serum is unlikely a cause for the poor recovery by RYSK173.

Although our study does not provide direct evidence that divalent metal ion binding to CN-1 masks the RYSK173 epitope, there seems to be a relation with trace metal ion concentrations in serum. Firstly, we found a significant difference in the proportion of RYSK173 in serum of patients with ESRD as compared to healthy controls. This was paralleled by a lower serum Zn^2+^ concentration in ESRD patients. Secondly, the proportion of RYSK173 CN-1 was decreased in the post-dialysis samples while in most patients the serum Zn^2+^ concentration was increased directly after dialysis. Although the Cu^2+^ concentration increased in parallel in the post-dialysis sample, direct binding of Cu^2+^ to the metal centres of CN-1 is highly unlikely.

A number of studies have suggested that CN-1 activity, either in serum or cerebrospinal fluid, might be important in the pathology of chronic kidney disease [20], diabetic complications [4, 8, 21], Alzheimer’s disease [22, 23] or dementia [24]. CN-1 activity is not only depending on CN-1 concentrations but also on the presence of competing substrates [1] and the relative proportion of CN-1 that is recognized by RYSK173 [10]. Although the RYSK173 is not intended for implementation in routine diagnostics, it might help understanding why in some patients CN-1 activity is extremely low despite the fact that CN-1 concentrations are normal.

In conclusion our study clearly indicates that RYSK173 recognizes a sequence within the transition metal binding site of CN-1, thus supporting our hypothesis that metal binding to CN-1 masks the epitope. The CN-1 RYSK173 proportion appears overall increased in ESRD patients which might be explained by a relative Zn deficiency. It remains to be assessed if Zn deficiency in general leads to an increase in the CN-1 RYSK173 proportion.
