# Correction: Monoclonal Antibody RYSK173 Recognizes the Dinuclear Zn Center of Serum Carnosinase 1 (CN-1): Possible Consequences of Zn Binding for CN-1 Recognition by RYSK173

**DOI:** 10.1371/journal.pone.0170919

**Published:** 2017-01-23

**Authors:** Shiqi Zhang, Holger A. Lindner, Sarah Kabtni, Jaap van den Born, Stephan Bakker, Gerjan Navis, Bernard K. Krämer, Benito Yard, Sibylle Hauske

The seventh author's name appears incorrectly in the published article. The correct name is: Bernhard K. Krämer.

## References

[1] Zhang S, Lindner HA, Kabtni S, van den Born J, Bakker S, Navis G, et al (2016) Monoclonal Antibody RYSK173 Recognizes the Dinuclear Zn Center of Serum Carnosinase 1 (CN-1): Possible Consequences of Zn Binding for CN-1 Recognition by RYSK173. PLoS ONE 11(1): e0146831 doi:10.1371/journal.pone.0146831 26799971 10.1371/journal.pone.0146831 PMC4723063

